# Effect of Dihydroartemisinin-Piperaquine on the Pharmacokinetics of Praziquantel for Treatment of *Schistosoma mansoni* Infection

**DOI:** 10.3390/ph14050400

**Published:** 2021-04-23

**Authors:** Omary Mashiku Minzi, Rajabu Hussein Mnkugwe, Eliford Ngaimisi, Safari Kinung’hi, Anna Hansson, Anton Pohanka, Appolinary Kamuhabwa, Eleni Aklillu

**Affiliations:** 1 Department of Clinical Pharmacy and Pharmacology, School of Pharmacy, Muhimbili University of Health and Allied Sciences, 11103 Dar es Salaam, Tanzania; minziobejayesu@gmail.com (O.M.M.); enali2012@gmail.com (A.K.); 2 Division of Clinical Pharmacology, Department of Laboratory Medicine, Karolinska Institutet, 141 86 Stockholm, Sweden; rajabuhussein06@gmail.com (R.H.M.); anton.pohanka@sll.se (A.P.); 3 Department of Clinical Pharmacology, School of Medicine, Muhimbili University of Health and Allied Sciences, 11103 Dar es Salaam, Tanzania; 4 Office of Clinical Pharmacology, Division of Pharmacometrics, Food and Drugs Administration, Silver Spring, MD 20993, USA; engaimisi@gmail.com; 5 National Institute for Medical Research, Mwanza Research Centre, 33104 Mwanza, Tanzania; kinunghi_csm@hotmail.com; 6 Department of Clinical Pharmacology, Karolinska University Hospital-Huddinge, 141 86 Stockholm, Sweden; anna.b.hansson@sll.se

**Keywords:** dihydroartemisinin-piperaquine, praziquantel, praziquantel enantiomers pharmacokinetics interaction, *Schistosoma mansoni*

## Abstract

Praziquantel (PZQ) and dihydroartemisinin-piperaquine (DHP) combination recently showed superior effectiveness than PZQ alone to treat intestinal schistosomiasis. In this follow-up study, we investigated the effect of DHP co-administration on the pharmacokinetics of PZQ and its enantiomers among 64 *Schistosoma mansoni* infected children treated with PZQ alone (*n* = 32) or PZQ + DHP combination (*n* = 32). Plasma samples collected at 0, 1, 2, 4, 6, and 8 h post-dose were quantified using UPLCMS/MS. The geometric mean (GM) of AUCs for total PZQ, R-PZQ and S-PZQ were significantly higher among children who received PZQ + DHP than PZQ alone. The geometric mean ratio (GMR) and (90% CI) of AUC_0–∞_ for PZQ + DHP to PZQ for total PZQ, R-PZQ, and S-PZQ were 2.18 (1.27, 3.76), 3.98 (2.27, 7.0) and 1.86 (1.06, 3.28), respectively. The GMR and (90% CI) of AUC_0–8_ for total PZQ, R-PZQ, and S-PZQ were 1.73 (1.12, 2.69), 2.94 (1.75, 4.92), and 1.50 (0.97, 2.31), respectively. The GM of C_max_ for total PZQ, R-PZQ and S-PZQ were significantly higher among those who received PZQ + DHP than PZQ alone. The GMR (90% CI) of C_max_ of PZQ + DHP to PZQ for total PZQ, R-PZQ, and S-PZQ were 1.75 (1.15, 2.65), 3.08 (1.91, 4.96), and 1.50 (1.0, 2.25%), respectively. The 90% CI of the GMRs for both AUCs and C_max_ for total PZQ, R-PZQ, and S-PZQ were outside the acceptable 0.80–1.25 range, indicating that the two treatment arms were not bioequivalent. DHP co-administration significantly increases systemic PZQ exposure, and this may contribute to increased effectiveness of PZQ + DHP combination therapy than PZQ alone to treat schistosomiasis.

## 1. Introduction

Schistosomiasis is a poverty-related parasitic infection that prevails in rural and disadvantaged populations in the tropics and sub-tropics regions [1,2]. It is estimated that more than 250 million people are infected with schistosomiasis globally [3,4]. A large proportion of the global burden is from children of Sub-Saharan Africa (SSA) [5,6]. Schistosomiasis is associated with anemia, poor growth, impaired physical fitness, and poor cognitive abilities in affected children [7,8,9,10] and delay in the achievement of Sustainable Development Goals (SDGs) [11,12].

Mass drug administration (MDA) using praziquantel (PZQ) targeting school-aged children is a World Health Organization (WHO)-recommended global strategy to control schistosomiasis in endemic settings [13]. Despite the use of mass PZQ treatment for several years, schistosomiasis remains a public health problem in most endemic countries in SSA, including Tanzania [8,14,15]. The lack of PZQ activity against the immature schistosome (schistosomula) is suggested as one of the reasons for the failure of MDA with PZQ in eliminating the disease as a public health problem [16]. Furthermore, an alarming threat of PZQ resistance in field studies from SSA has been previously reported [17]. Therefore, there is pressing need for a broad anthelminthic approach supplementing PZQ with new antischistosomal drugs that targets different parasite developmental stages and/or with a different mechanism of action [18], to increase treatment efficacy and reduce the risk of PZQ resistance [19]. 

Combination of drugs with different mechanisms of action has become a common strategy to increase efficacy and reduce or delay the development of drug resistance. Previous studies indicated that artemisinin derivatives have capacity to kill immature schistosomes hence improving the efficacy of PZQ [16,20,21]. In a randomized clinical trial, we recently reported superior effectiveness of PZQ plus dihydroartemisinin-piperaquine (DHP) combination therapy targeting to kill both mature and immature parasites than PZQ alone for the treatment of schistosomiasis in infected children [22]. As the use of combination of drugs increases, the likelihood of drug interactions, any effect of DHP on the pharmacokinetics of PZQ and its enantiomers needs to be investigated. Most pharmacokinetic drug interactions occur at a metabolic level altering drug metabolism and disposition. Dihydroartemisinin (DHA), an artemisinin derivative, is mainly eliminated via glucuronidation by UDP-glucuronosyltransferases (UGTs) UGT1A9 and UGT2B7 [23]. Both PZQ and piperaquine (PPQ, an aminoquinoline derivative) are metabolized by cytochrome P450 (CYP450) enzymes mainly via CYP3A4 and this may pose a risk of drug–drug interactions [24,25]. Chloroquine (CHQ), which is also an aminoquinoline derivative like PPQ [26] is reported to reduce the bioavailability and maximum concentration (C_max_) of PZQ in humans despite the non-competitive inhibition on PZQ metabolism [27]. Therefore, the likelihood of drug–drug interactions of clinical relevance between PZQ and DHP when co-administered for treatment of schistosomiasis warrant further investigation. 

PZQ is a racemic mixture of R-praziquantel (R-PZQ) and S-praziquantel (S-PZQ) enantiomers, and recent findings reported higher antischistosomal activity of R-PZQ enantiomer when administered alone compared to the racemate at the same dose [28,29]. Overall data on the pharmacokinetics profile of PZQ and its enantiomers among infected children is scarce, and any potential drug interactions of PZQ and DHP combination therapy albeit increased efficacy, remains to be investigated [22]. This study compared the pharmacokinetic profiles of PZQ and its enantiomers when PZQ is given alone or in combination with DHP for treatment of *Schistosoma mansoni* infection among school children in North-Western Tanzania.

## 2. Results

### 2.1. Sociodemographic and Baseline Characteristics

A total of 64 *Schistosoma mansoni* infected children (32 received PZQ alone and 32 received PZQ + DHP) were enrolled and completed this study. The mean age (±SD) of the study population was 12.7 ± 1.8 years. Females were 45.3% (29/64) of all study participants. There was no significant difference in patient’s and baseline characteristics between treatment arms (*p* > 0.05) (Table 1). 

### 2.2. Effect of DHP on the Pharmacokinetic Parameters of Total PZQ

The arithmetic and geometric means of AUC_0–8_ and AUC_0–∞_ for total PZQ were significantly higher among children who received PZQ + DHP combination than those who received PZQ alone (Table 2). The final linear mixed effect (LME) models included treatment arm (*p* = 0.028) and Body Mass Index (BMI) (*p* = 0.042) as statistically significant predictors of total PZQ C_max_; treatment arm (*p* = 0.041) and BMI (*p* = 0. 021) as statistically significant predictors of total PZQ AUC_0–8_; treatment arm (*p* = 0.021) and BMI (*p* = 0. 079) as predictors of total PZQ AUC_0–∞_; and treatment arm (*p* = 0.007) and sex (*p* = 0. 012) as statistically significant predictors of total PZQ half-life. 

**Figure 1:**
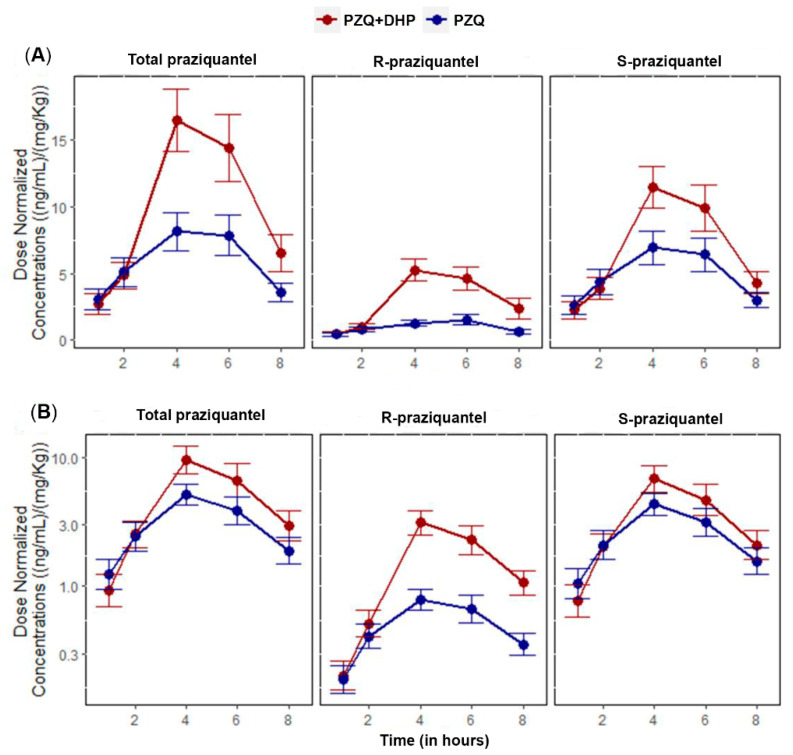
Comparisons of mean of dose-normalized concentration-time profiles of total PZQ, R-PZQ and S-PZQ between PZQ versus PZQ + DHP treatment arms. Error bars represent standard errors of the mean (SEM). (**A**) Linear/linear scale (**B**) Log/linear scale.

**Figure 2:**
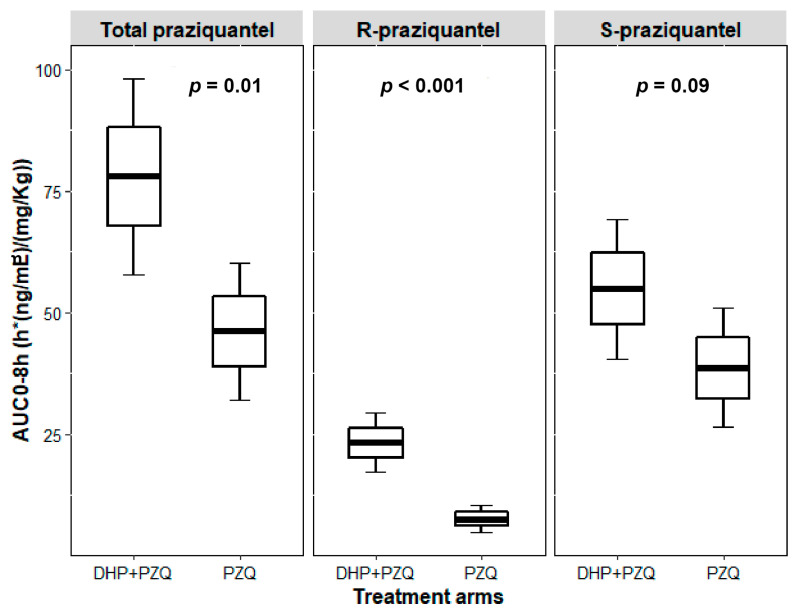
Boxplots comparing dose-normalized AUC_0–8h_ for total PZQ, R-PZQ and S-PZQ between PZQ versus PZQ + DHP treatment arms. Boxes represent mean ± SE, error bars represent mean ± 1.96*SE.

The AUC_0–∞_ geometric mean ratio (GMR) of PZQ + DHP to PZQ for total PZQ was 2.18 and 90% CI of 1.27–3.76. For AUC_0–8_, the GMR was 1.73 and 90% CI of 1.12–2.69. The 90% CI of GMRs for both AUC_0–∞_ and AUC_0–8_ of total PZQ were outside the bioequivalence limits of 0.80–1.25. Infected children who received PZQ + DHP combination had significantly higher total PZQ systemic exposure than those who received PZQ alone. Comparison of the mean dose-normalized concentration-time profile and box plot of AUC_0–8_ for total PZQ in the presence and absence of DHP is presented in Figure 1 and Figure 2, respectively. The arithmetic and geometric means of C_max_ of total PZQ were significantly higher among children who received PZQ + DHP combination than those who received PZQ alone. The GMR of PZQ + DHP to PZQ of the C_max_ was 1.75 and 90% CI of 1.15–2.65 (Table 2). The observed 90% CI of the GMR for C_max_ for total PZQ was also outside the acceptable range of 0.80–1.25. There was no significant effect of DHP on the elimination half-life (hours) and time to reach maximum plasma concentration (T_max_ in hours) of total PZQ (Table 2). The arithmetic means (±SD) and medians (IQR) of the pharmacokinetics parameters for total PZQ are presented in the supplementary file (Table S1).

### 2.3. Effect of DHP on the Pharmacokinetic Parameters of R-PZQ and S-PZQ

Like what was observed with total PZQ, the arithmetic and geometric means of AUC_0–8_ and AUC_0–∞_ for R-PZQ and S-PZQ were significantly higher among children who received PZQ + DHP combination than those who received PZQ alone (Table 3). The arithmetic and geometric means of the AUCs and C_max_ of S-PZQ were higher than the one for R-PZQ (Table 3). 

The final LME models for R-PZQ included treatment arm (*p* < 0.001) only as a statistically significant predictor of R-PZQ C_max_; treatment arm (*p* < 0.001) only as a statistically significant predictor of R-PZQ AUC_0–8_; treatment arm (*p* < 0.001) only as statistically significant predictor of R-PZQ AUC_0–∞_; and treatment arm (*p* = 0.074) as predictors of R-PZQ half-life. The final LME models for S-PZQ included treatment arm (*p* = 0.102) and BMI (*p* = 0.022) as significant predictors of S-PZQ C_max_; treatment arm (*p* = 0.123) and BMI (*p* = 0.009) as predictors of S-PZQ AUC_0–8_; treatment arm (*p* = 0.072) and BMI (*p* = 0.052) as predictors of S-PZQ AUC_0–∞_; and treatment arm (*p* = 0.035) and sex (*p* = 0. 047) as predictors of S-PZQ half-life.

For R-PZQ, the GMR of PZQ + DHP to PZQ for AUC_0–∞_ was 3.98 and 90% CI of 2.27–7.00. For AUC_0–8_, the GMR was 2.94 and 90% CI of 1.75–4.92. The 90% CI of the GMRs for AUC_0–∞_ and AUC_0–8_ were outside bioequivalence limits of 0.80–1.25. The arithmetic and geometric means of C_max_ for R-PZQ were significantly higher among children who received PZQ + DHP combination than those who received PZQ alone. The GMR of PZQ + DHP to PZQ of the C_max_ for R-PZQ was 3.08 and 90% CI of 1.91–4.96 (Table 3). The 90% CI of the GMR for C_max_ like AUCs was also outside the bioequivalence limits of 0.80–1.25. 

For S-PZQ, the GMR of PZQ + DHP to PZQ for AUC_0–∞_ was 1.86 and 90% CI of 1.06–3.28. For AUC_0–8_, the GMR was 1.50 and 90% CI of 0.97–2.31. Similar to R-PZQ, the 90% CI of the GMRs for both AUC_0–∞_ and AUC_0–8_ for S-PZQ were outside the bioequivalence limits of 0.80–1.25. The arithmetic and geometric means of C_max_ for S-PZQ were higher among children who received PZQ + DHP combination than those who received PZQ alone. The GMR of PZQ + DHP to PZQ of the C_max_ for S-PZQ was 1.50 and 90% CI of 1.0–2.25 (Table 3). The 90% CI of the GMR for C_max_ was also outside the bioequivalence limits of 0.80–1.25. Comparison of mean dose-normalized concentration-time profile and box plot of AUC_0–8_ for R-PZQ and S-PZQ in the presence and absence of DHP are presented in Figure 1 and Figure 2, respectively. There was no significant effect of DHP on elimination half-life (hours) and T_max_ in hours of both R-PZQ and S-PZQ (Table 3). The arithmetic means (±SD) and medians (IQR) of the pharmacokinetics parameters for R-PZQ and S-PZQ are presented in the supplementary file (Tables S2 and S3).

## 3. Discussion

In a randomized clinical trial, we recently reported that PZQ and DHP combination therapy is safe and more efficacious than PZQ alone for the treatment of intestinal schistosomiasis in infected children [22]. Drugs co-administered with PZQ may have a similar CYP450 metabolic pathway and potentially alter PZQ systemic exposure, which may in turn influence treatment outcomes. This follow-up pharmacokinetic drug-interaction study investigated the effect of DHP on the pharmacokinetic profile of total PZQ and its enantiomers among *Schistosoma mansoni* infected school children. Our results indicate a significant drug–drug interaction between DHP and PZQ, in which systemic drug exposure increased among infected children treated with PZQ + DHP combination therapy than those who received PZQ monotherapy as reflected by the increased AUC_s_ and C_max_. The change in AUC of a drug, representing its systemic exposure, is the main parameter considered in the evaluation of drug–drug interactions (DDIs) [30]. To our knowledge, this is the first study to investigate drug interaction between DHP and PZQ and its clinical relevance in *Schistosoma mansoni* infected children. 

Our result indicates significantly higher AUC_0–∞_, AUC_0–8_ and C_max_ of both total PZQ and its enantiomers R-PZQ and S-PZQ among children treated with PZQ + DHP combination therapy than those who received PZQ alone. The 90% CI of the geometric mean ratios of AUCs and C_max_ for both total PZQ, R-PZQ, and S-PZQ were outside the acceptable bioequivalent interval of 0.80–1.25 (Table 2 and Table 3), indicating that systemic exposure of PZQ in the presence and absence of DHP is not bioequivalent. 

In the combination therapy arm, participants received PZQ with a fixed dose dihydroartemisinin and piperaquine combination (DHP). Dihydroartemisinin is eliminated via glucuronidation catalyzed by UDP-glucuronosyltransferases, mainly by UGT1A9 and UGT2B7 [23]. Both PZQ and piperaquine undergo metabolism via cytochrome P450 enzymes, primarily by CYP3A4 [24,25]. Based on their metabolic pathways, the observed significant increase in plasma PZQ exposure is most likely due to competitive inhibition of CYP3A4/5 enzymes by piperaquine but not by dihydroartemisinin. CYP3A-mediated drug interaction is a possible mechanism by which DHP co-administration increases systemic PZQ exposure as reflected by significantly high AUC_0–∞_, AUC_0–8_, and C_max_ for total PZQ and its enantiomers among children who received PZQ + DHP combination therapy. A similar inhibition effect on PZQ metabolism by another aminoquinoline drug chloroquine is reported previously [26,27].

Using a randomized clinical trial, we recently reported a significantly higher cure rate both at 3- and 8-weeks post-treatment in *Schistosoma mansoni* infected children who were treated with PZQ + DHP combination therapy compared to PZQ alone [22]. This follow-up drug-interaction study reveals that co-administration of DHP significantly increases plasma exposure and bioavailability of PZQ. In pharmacokinetics-pharmacodynamics (PK-PD) studies, high plasma drug concentration is used as a surrogate for a high drug concentration in tissue. Accordingly, higher plasma PZQ systemic exposure by DHP co-administration can enhance PZQ therapeutic efficacy and increased the cure rate. Indeed, PZQ is reported to show dose-dependent treatment response in school-aged children, increasing cure rates and egg reduction rates with escalating dosages of PZQ [31]. A previous PZQ PK-PD study reported that higher total PZQ AUC_s_ were associated with an increased parasitological cure, and a higher PZQ dose is required for maximal efficacy [32]. PZQ + DHP combination therapy is advantageous not only for killing both mature and immature stages of the parasite [18] and protection against malaria [33] but also in increasing systemic PZQ exposure and its therapeutic efficacy. Thus, the reported higher efficacy among the children treated with PZQ + DHP combination therapy could possibly be due to both higher systemic PZQ exposure and the killing of immature schistosomes by DHP. 

PZQ administration has two major drawbacks, the first being the high dose needed (40 mg/kg body weight) and the second is its bitter and unpleasant taste [34]. Younger children usually cannot swallow the existing tablets (600 mg) because of their large size and bitter taste, causing compliance problems. Apart from increasing efficacy, the observed significant DDIs in the PZQ + DHP combination therapy can possibly alleviate the drawbacks of PZQ monotherapy. Our result indicates that the inhibitory effect of DHP is more pronounced for R-PZQ than the S-PZQ (Figure 2), and this can be an advantage for the combination therapy since R-PZQ is the therapeutically active enantiomer and has less bitter taste compared to racemic PZQ [34]. Therefore PZQ-DHP combination therapy not only offers the chance to reduce PZQ dose, but also the potential to reduce the pill burden and the unpleasant taste of the racemic PZQ. Synergistic and additive drug–drug interactions with the intention to reduce the dose of PZQ while still having an efficacious effect using the antimalarial mefloquine was explored in rodents previously [35]. A similar approach can be investigated for PZQ-DHP combination in humans.

Higher PZQ systemic drug exposure may result in not only increased effectiveness but also occurrence of adverse events among those treated with PZQ + DHP combination therapy. However, no significant difference in the overall occurrence of adverse events was reported between children treated with PZQ + DHP combination and PZQ alone [22]. Therefore, an increased systemic PZQ exposure by co-administration of DHP without affecting safety, yet improved efficacy, is an important finding which complements the current standard treatment and can serve as an alternative regimen to PZQ monotherapy.

## 4. Materials and Methods

### 4.1. Study Design, Area, and Population

This was a parallel two-arm pharmacokinetic study to investigate potential pharmacokinetic drug interaction between PZQ and DHP and its clinical relevance. This study was part of a randomized clinical trial registered with PACTR201612001914353 [22]. A total of 64 *Schistosoma mansoni* infected school children aged 9–17 years old attending Fogofogo Primary School in Busega district, Simiyu region, North-Western Tanzania who participated in our previous randomized clinical trial [22] were included in this follow-up pharmacokinetic drug-interaction study. The study area was a rural village located along the shores of Lake Victoria endemic for intestinal schistosomiasis.

### 4.2. Treatment and Samples Collection for Pharmacokinetic Study

Pre-treatment standardized meal was given to study participants as reported previously [22,36], as food has been known to increase PZQ bioavailability [37,38]. A total of 64 *Schistosoma mansoni* infected children were treated with either single-dose PZQ 40 mg/kg bodyweight (*n* = 32) alone or PZQ + DHP combination therapy (*n* = 32). The dose of DHP tablets (40 mg dihydroartemisinin / 320mg piperaquine) was calculated based on the child’s body weight (in kg) and was given once per day for three consecutive days following the WHO treatment guideline for uncomplicated malaria [39]. PZQ and the first dose of DHP were administered at zero hour. Two mL of venous blood was collected at 0, 1, 2, 4, 6 and 8 h post-dose from the antecubital arm vein using an indwelling intravenous catheter into heparinized vacutainer tubes. Blood samples were centrifuged at 1000 rpm for 10 min to obtain plasma stored at −80 °C freezer for drug quantification. 

### 4.3. Chemicals and Reagents

**Figure 3:**
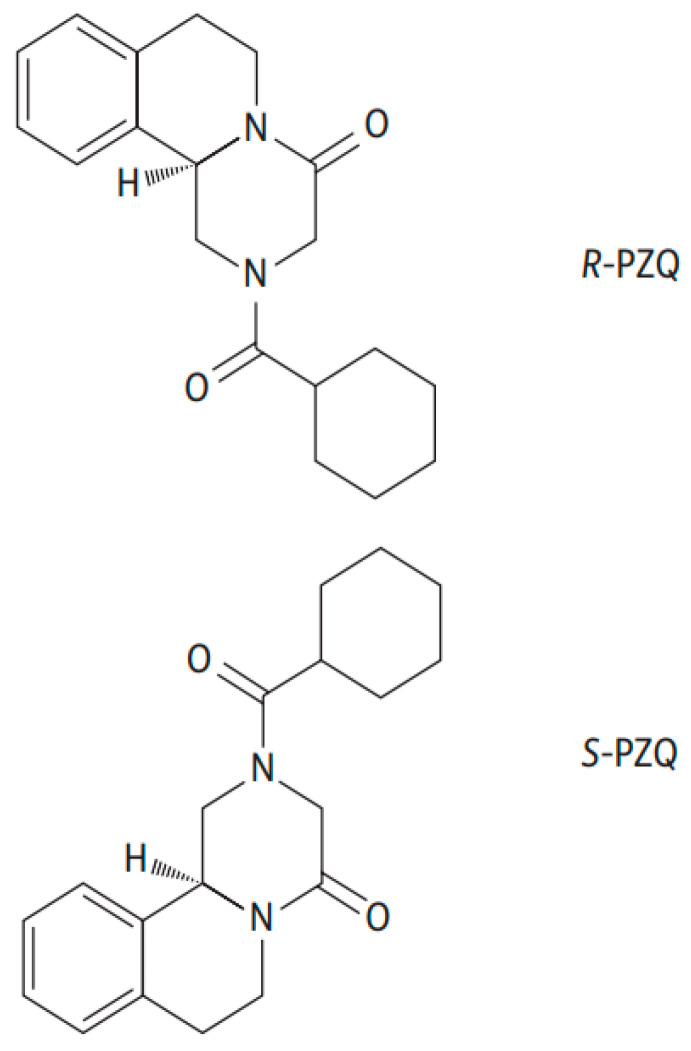
The chemical structure of R-PZQ and S-PZQ enantiomers [38].

R-PZQ, S-PZQ and rac-PZQ were purchased from Merck (Darmstadt, Germany). An eleven-fold rac-deuterated-PZQ (rac-PZQ-d11) as an internal standard (IS) was purchased from Toronto Research Chemicals (Toronto, Ontario, Canada). Figure 3 presents the chemical structure of R-PZQ and S-PZQ [38]. Acetone, acetonitrile, ammonium acetate, isopropanol, methanol, and acetic acid of mass spectrometry (MS) grade were purchased from Merck (Darmstadt, Germany). Deionized water/Milli-Q water/ultrapure water was prepared using a Milli-Q water purification system (Merck Millipore, MA, USA). Blank human plasma was kindly supplied by the blood bank of the Karolinska University Hospital Huddinge (Stockholm, Sweden).

### 4.4. Analytical Method and Validation

A two-channel system consisting of a Dionex Ultimate 3000RS liquid chromatography (LC) system with a TriPlus RSI autosampler and a Thermo Scientific TSQ Quantis triple quadrupole mass spectrometer (MS/MS) was used to quantify R-PZQ and S-PZQ. The MS/MS was operated in positive ionization mode. The data were processed using TraceFinder 4.1 (Thermo Scientific). The LC-MS/MS method for analysis of R-PZQ and S-PZQ was adapted from a recently validated enantioselective method described by Kovac et al. [28]. Briefly, plasma calibration samples were freshly prepared by spiking blank plasma samples with rac-PZQ and were included in each analytical run. Quality control samples were also prepared by spiking plasma blanks to obtain low, medium, and high concentrations for both R-PZQ and S-PZQ. The quantification range of the method was 1 to 1500 ng/mL for both R-PZQ and S-PZQ.

For extraction of analytes of interest, 100 µL of plasma samples went through protein precipitation with 200 µL of internal standard solution (50 ng/mL of rac-PZQ-d11 in methanol) and then vortexed for 10 s followed by centrifugation for 5 min at 2100× *g*. 150 µL of the supernatant was diluted with 75 µL Milli-Q water and 5 µL was injected onto the LC-MS/MS system. The chromatographic system was using a Chiralpak AGP 2.0 × 100 mm, 5 µm, column (Chiral Technologies Europe, Illkirch, France) with 10 mM ammonium acetate: isopropanol 98:2 (*v*/*v*) pH 8 as mobile phase with a flow rate of 0.3 mL/min. The chromatographic run was 22 min, and with use of the parallel two-channel capacity, injection to injection time was 11 min. R-PZQ eluted first followed by S-PZQ with a difference of 1.9 min.

Praziquantel was monitored by the transition m/z 313.2 > 202.9 and the IS rac-PZQ-d11by 324.2 > 204.1. The analytical method was validated according to the European Medicines Agency Guideline on bioanalytical method validation [40]. The calibration curve was constructed by linear regression of the analyte/internal standard area ratios with an applied weighing of 1/x^2^. Accuracy was within ±5% except for lower limit of quantification (LLOQ) where it was within ±12%. Precision was below 5 CV% except for LLOQ (below 13 CV%).

### 4.5. Pharmacokinetic Data Analysis

Noncompartmental analysis (NCA) with linear trapezoidal rule was used to calculate pharmacokinetics parameters using R statistical software version 4.0.2 [41]. The primary and secondary pharmacokinetic parameters include maximum plasma concentration (C_max_) in ng/mL, time needed to reach C_max_ (T_max_) in hours, area under the concentration—time curve from zero hour to infinity (AUC_0–∞_, ng·h/mL), area under the concentration—time curve from zero hour to 8 h post-dose (AUC_0–8h_, ng·h/mL) and terminal half-life (t_1/2_) in hours for R-PZQ, S-PZQ, and total PZQ. C_max_, T_max_, t_1/2_, AUC_0–8h_, and AUC_0–∞_ were calculated from the pharmacokinetics raw data using the PKNCA package version 0.9.4 implemented in R [42].

### 4.6. Statistical Data Analysis

Summary statistics of the pharmacokinetic parameters for each treatment group were reported as arithmetic mean, standard deviation (SD), geometric mean and coefficient of variation (CV %). The geometric mean is a mean or average which indicates the central tendency of the data that have been log 10-transformed before statistical analysis (e.g., AUC or C_max_) and calculated as antilogarithm of the mean of the log 10 transformed data. Coefficient of variation (CV %) was calculated using the formula $\mathrm{CV}\%=\sqrt{e^{\mathrm{Variance}\;\mathrm{of}\;X}-1}$ where X is log-transformed pharmacokinetic parameter values. Concentrations, AUCs and C_max_ were dose-normalized by dividing concentration or pharmacokinetics parameter values by received dose. Independent *t*-test was used to compare the means of the log-transformed AUC_0–8_ between treatment groups.

Linear mixed effect modeling was used to determine subject-characteristics that influence noncompartmental pharmacokinetic parameters values (C_max_, AUC_0–8_, AUC_0–∞_, and t_1/2_) of R-PZQ, S-PZQ, and total PZQ. The evaluated subject-characteristics for each pharmacokinetic parameter included age, weight, BMI, treatment arm (PZQ + DHP vs PZQ) and sex. For each pharmacokinetic parameter, a model of log-transformed values versus treatment arm was built as a base model. Additional subject-characteristics were added individually to the base model in a stepwise manner. An added subject-characteristic was retained in the model if it was found to be significantly associated with the pharmacokinetic parameter (*p* < 0.05). The final modes were parameterized as follows: $\ln\left(\mathrm{PK}\right)∼\beta \times \mathrm{TRT}+\alpha_{i}\times {\;\mathrm{CHAR}}_{i}+\varepsilon$, where PK represents pharmacokinetic parameters, β represents fixed effect parameter for the treatment arm (PZQ being the reference arm), $\alpha_{i}$ represents fixed effect parameter for additional subject-characteristics (${\mathrm{CHAR}}_{i}$), and ε represents between-subject variability of the pharmacokinetics. Parameters of the final models were used to calculate the geometric mean ratio (GMR) of the test (PZQ + DHP) to reference (PZQ) treatment arms and corresponding 90% confidence intervals (CI). In brief, GMR was calculated as antilogarithm of β, while 90% CI were calculated using the final model ε in the test arm, β, degrees of freedom in the test arm (*n* = 28) and alpha value of 0.05. The 90% confidence interval (CI) of the GMRs for AUCs and C_max_ were calculated to assess bioequivalence between PZQ + DHP versus PZQ alone treatment. Drug–drug interaction was concluded when the 90% CI of GMR of PZQ + DHP to PZQ ratio for a pharmacokinetic parameter was not entirely contained within the acceptable bioequivalence limits of 0.80–1.25 (no effect boundaries). 

## 5. Conclusions

In summary, DHP co-administration significantly increases systemic exposure of total PZQ and its enantiomers. Our study provides a new insight in which CYP3A-mediated drug interaction as a possible contributor for the increased effectiveness of the PZQ + DHP combination therapy than PZQ monotherapy for the treatment and control of schistosomiasis. DHP+PZQ combination therapy is beneficial not only to kill both mature and immature stages of the parasites but also increases PZQ bioavailability and its therapeutic efficacy. The synergistic and additive drug–drug interactions between DHP and PZQ without compromising safety makes DHP+PZQ combination therapy as a better alternative than PZQ alone for MDA in schistosomiasis control and elimination program. However, any effect of PZQ co-administration on DHP pharmacokinetics needs further investigation. 

## Figures and Tables

**Table 1:** Sociodemographic and baseline characteristics of study participants.

Variable		Treatment Arm		*p*-Value
		PZQ	PZQ + DHP	
Age (years)	Mean ± SD Range	12.8 ± 1.6 10–17	12.7 ± 2.0 9–16	0.79 ^a^
Sex	Male N (%)	17 (53.1)	18 (56.3)	0.85
	Female N (%)	15 (46.9)	14 (43.8)	0.87
Weight (kg)	Mean ± SD	33.1 ± 6.2	33.7 ± 9.3	0.74 ^a^
Height (cm)	Mean ± SD	141.0 ± 9.7	143.9 ± 12.9	0.32 ^a^
Haemoglobin (g/dL)	Mean ± SD	12.4 ± 1.7	12.2 ± 1.9	0.71 ^a^
Infection intensity	Eggs per gran Median (range)	246 (12–1452)	165 (6–1722)	0.32 ^b^
	Light N (%)	9 (28.1)	14 (43.8)	0.46
	Moderate N (%)	11 (34.4)	8 (25.0)	0.67
	Heavy N (%)	12 (37.5)	10 (31.3)	0.77

^a^—Independent Student *t*-test; ^b^—Manny Whitney U test; SD—Standard deviation.

**Table 2:** Comparisons of pharmacokinetic parameters of total PZQ between PZQ + DHP and PZQ treatment arms.

Parameters	Treatment Arm		(PZQ + DHP)|PZQ
	PZQ + DHP	PZQ	
	Geometric mean (CV %)	Geometric mean (CV %)	Geometric Mean Ratio (90% CI)
AUC (0-Inf)	66.8 (112.1)	33.5 (106.4)	2.18 (1.27–3.76)
AUC (0–8h)	50.4 (181.8)	32.4 (111.9)	1.73 (1.12–2.69)
C_max_ (ng/mL)	14.3 (169)	8.9 (95.1)	1.75 (1.15–2.65)
Half-life (h)	1.5 (29.7)	2.2 (47.5)	0.7 (0.57–0.86)
T_max_ (h)	3.6 (55.7)	3.5 (61.8)	

CV%—coefficient of variation; IQR—Interquartile range.

**Table 3:** Comparisons of pharmacokinetic parameters of R-PZQ and S-PZQ between PZQ + DHP and PZQ treatment arms.

Parameters	R-PZQ		(PZQ + DHP)|PZQ GMR (90% CI)	S-PZQ		(PZQ + DHP)|PZQ GMR (90% CI)
	Treatment Arm			Treatment Arm		
	PZQ + DHP GM (CV%)	PZQ GM (CV%)		PZQ + DHP GM (CV%)	PZQ GM (CV%)	
AUC (0-Inf)	19.5 (118.1)	4.9 (84.2)	3.98 (2.27–7.0)	44.8 (114.6)	27.1 (119.6)	1.86 (1.06–3.28)
AUC (0–8h)	14.2 (235.3)	4.8 (144.1)	2.94 (1.75–4.92)	35.6 (174.9)	26.7 (114.9)	1.5 (0.97–2.31)
C_max_ (ng/mL)	4.5 (206.5)	1.5 (119.5)	3.08 (1.91–4.96)	9.9 (160.1)	7.3 (96.3)	1.5 (1.0–2.25)
Half-life (h)	1.6 (34.3)	2 (35.9)	0.79 (0.63–0.98)	1.7 (26.7)	2.1 (43.5)	0.77 (0.63–0.94)
T_max_ (h)	3.8 (51.4)	3.5 (62.8)		3.6 (55.2)	3.5 (61.8)	

CV—Coefficient of variation; GM—Geometric mean.

## Data Availability

All data presented in this study are contained within the manuscript and its supporting file.

## Supplementary Materials

The following are available online at https://www.mdpi.com/article/10.3390/ph14050400/s1, Table S1: Comparisons of pharmacokinetic parameters of total PZQ between PZQ + DHP and PZQ treatment arms, Table S2: Comparisons of pharmacokinetic parameters of R-PZQ between PZQ + DHP and PZQ treatment arms, Table S3: Comparisons of pharmacokinetic parameters of S-PZQ between PZQ + DHP and PZQ treatment arms.
