# Supplementary material for: Effect of Dihydroartemisinin-Piperaquine on the Pharmacokinetics of Praziquantel for Treatment of Schistosoma mansoni Infection
Source: Pharmaceuticals (Basel). 2021 Apr 23;14(5):400. doi: 10.3390/ph14050400 (PMC8145331; doi:10.3390/ph14050400)
Supplement: Supplementary file 1 [file pharmaceuticals-14-00400-s002.zip › pharmaceuticals-1181015-supplementary.pdf]

Table S1: Comparisons of pharmacokinetic parameters of total PZQ between PZQ+DHP and PZQ treatment arms

Parameters	PZQ + DHP			PZQ			(PZQ+DHP)/PZQ
	Mean (SD)	Median (IQR)	Geometric mean (CV %)	Mean (SD)	Median (IQR)	Geometric mean (CV %)	Geometric Mean Ratio (90% CI)
**AUC (0-Inf)**	93.7 (74.4)	87.8 (30.3 - 130.7)	66.8 (112.1)	49.9 (58)	34.5 (17.6 - 48.9)	33.5 (106.4)	2.18 (1.27 - 3.76)
**AUC (0-8h)**	77.9 (57.9)	66.9 (27.8 - 114.5)	50.4 (181.8)	46.1 (40.2)	32.9 (20.7 - 65.4)	32.4 (111.9)	1.73 (1.12 - 2.69)
**Cmax (ng/mL)**	21.3 (14.4)	20.2 (7.2 - 31.8)	14.3 (169)	11.9 (9.1)	9.1 (6 - 14.8)	8.9 (95.1)	1.75 (1.15 - 2.65)
**Half-life (h)**	1.6 (0.5)	1.6 (1.2 - 1.9)	1.5 (29.7)	2.5 (1.7)	1.9 (1.7 - 2.3)	2.2 (47.5)	0.7 (0.57 - 0.86)
**Tmax (h)**	4 (1.6)	4 (4 - 4)	3.6 (55.7)	4 (1.7)	4 (2 - 6)	3.5 (61.8)	-

CV % - coefficient of variation; IQR – Interquartile range

Table S2: Comparisons of pharmacokinetic parameters of R-PZQ between PZQ+DHP and PZQ treatment arms

Parameters	PZQ + DHP			PZQ			(PZQ+DHP)/PZQ
	Mean (SD)	Median (IQR)	Geometric mean (CV %)	Mean (SD)	Median (IQR)	Geometric mean (CV %)	Geometric Mean Ratio (90% CI)
**AUC (0-Inf)**	27.5 (21)	25.4 (9.8 - 37.4)	19.5 (118.1)	6.3 (5)	5.5 (3 - 6.3)	4.9 (84.2)	3.98 (2.27 – 7.0)
**AUC (0-8h)**	23.3 (17.3)	17.6 (9.9 - 36.7)	14.2 (235.3)	7.6 (7.7)	4.9 (2.5 - 8.4)	4.8 (144.1)	2.94 (1.75 - 4.92)
**Cmax (ng/mL)**	7.4 (5.8)	6.6 (2.3 - 10.8)	4.5 (206.5)	2.2 (2.1)	1.5 (0.7 - 2.4)	1.5 (119.5)	3.08 (1.91 - 4.96)
**Half-life (h)**	1.6 (0.7)	1.5 (1.2 - 1.8)	1.6 (34.3)	2.1 (0.7)	1.9 (1.6 - 2.5)	2 (35.9)	0.79 (0.63 - 0.98)
**Tmax (h)**	4.1 (1.6)	4 (4 - 4.5)	3.8 (51.4)	4 (1.8)	4 (2 - 6)	3.5 (62.8)	

Table S3: Comparisons of pharmacokinetic parameters of S-PZQ between PZQ+DHP and PZQ treatment arms

Parameters	PZQ + DHP			PZQ			(PZQ+DHP)/PZQ
	Mean (SD)	Median (IQR)	Geometric mean (CV %)	Mean (SD)	Median (IQR)	Geometric mean (CV %)	Geometric Mean Ratio (90% CI)
**AUC (0-Inf)**	64.7 (56.8)	56.8 (19.8 - 88.9)	44.8 (114.6)	43 (53.8)	26.5 (14.4 - 43.3)	27.1 (119.6)	1.86 (1.06 - 3.28)
**AUC (0-8h)**	54.8 (41.6)	50 (19 - 78.2)	35.6 (174.9)	38.6 (34.7)	25.6 (16.1 - 56.5)	26.7 (114.9)	1.5 (0.97 - 2.31)
**Cmax (ng/mL)**	14.5 (9.7)	15.4 (5.1 - 21.2)	9.9 (160.1)	9.7 (7.4)	7.5 (4.6 - 12.7)	7.3 (96.3)	1.5 (1.0 - 2.25)
**Half-life (h)**	1.7 (0.5)	1.8 (1.4 - 1.9)	1.7 (26.7)	2.3 (1.5)	1.8 (1.8 - 2.3)	2.1 (43.5)	0.77 (0.63 - 0.94)
**Tmax (h)**	4 (1.5)	4 (4 - 4.5)	3.6 (55.2)	4 (1.7)	4 (2 - 6)	3.5 (61.8)	
